# BDNF Influence on Adult Terminal Axon Sprouting after Partial Deafferentation

**DOI:** 10.3390/ijms241310660

**Published:** 2023-06-26

**Authors:** Beatriz Benítez-Temiño, Rosendo G. Hernández, Rosa R. de la Cruz, Angel M. Pastor

**Affiliations:** Departamento de Fisiología, Facultad de Biología, Universidad de Sevilla, 41012 Seville, Spain

**Keywords:** neurotrophin, reactive synaptogenesis, oculomotor system, motoneuron

## Abstract

BDNF is a neurotrophin family member implicated in many different neuronal functions, from neuronal survival during development to synaptic plasticity associated with processes of learning and memory. Its presence in the oculomotor system has previously been demonstrated, as it regulates afferent composition of extraocular motoneurons and their firing pattern. Moreover, BDNF expression increases after extraocular motoneuron partial deafferentation, in parallel with terminal axon sprouting from the remaining axons. To elucidate whether BDNF could play an active role in this process, we performed partial deafferentation of the medial rectus motoneurons through transection of one of the two main afferents, that is, the ascending tract of Deiters, and injected BDNF into the motoneuron target muscle, the medial rectus. Furthermore, to check whether BDNF could stimulate axon sprouting without lesions, we performed the same experiment without any lesions. Axon terminal sprouting was assessed by calretinin immunostaining, which specifically labels the remaining afferent system on medial rectus motoneurons, the abducens internuclear neurons. The results presented herein show that exogenous BDNF stimulated terminal axon growth, allowing the total recovery of synaptic coverage around the motoneuron somata. Moreover, calretinin staining in the neuropil exceeded that present in the control situation. Thus, BDNF could also stimulate axonal sprouting in the neuropil of intact animals. These results point to an active role of BDNF in plastic adaptations that take place after partial deafferentation.

## 1. Introduction

Reactive synaptogenesis has been described as the process by which, when a population of neurons is partially disconnected from its afferents, the remaining terminal inputs sprout and occupy the vacant synaptic spaces [1,2,3,4,5,6,7]. This process has been described, among others, in the visual [1] and vestibular systems [5], spinal cord [6], and hippocampus [2,3,4].

In recent years, reactive synaptogenesis has also been described in the oculomotor system [7,8]. Medial rectus motoneurons are located in the midbrain oculomotor nucleus, and project through the third cranial nerve to the ipsilateral medial rectus muscle to produce adducting eye movements in the horizontal plane [9]. This population of motoneurons receives two main inputs from two different pontine locations. First, the abducens nucleus contains two different populations of neurons: the lateral rectus motoneurons and the abducens internuclear neurons, which receive the same inputs as abducens motoneurons, and project through the contralateral medial longitudinal fascicle (MLF) to innervate the contralateral medial rectus motoneurons [10,11,12]. Second, the ipsilateral vestibular neurons in the lateral vestibular nucleus project through the ipsilateral ascending tract of Deiters (ATD), parallel and lateral to the MLF, to establish synaptic contacts with medial rectus motoneurons [13,14,15]. There is a partial anatomical segregation of these two different inputs, since abducens internuclear neurons mainly innervate medial rectus motoneuron dendrites, while vestibular inputs preferentially terminate in the somata [16]. Thus, partial deafferentation of the medial rectus motoneuron, either by MLF or ATD section, represents a good model for analysing the process of reactive synaptogenesis.

When an ATD section is performed, a distal axon degeneration is already visible 24 h after lesion. While this degenerative process continues, the remaining input population, i.e., abducens internuclear neuron terminals, start to sprout and form new terminal boutons that are visible 96 h post-lesion around both the dendrites and the soma of medial rectus motoneurons [8]. An increase in the axon growth-associated protein GAP-43 has been described in the neuropil surrounding medial rectus motoneurons, pointing to a reactivation of the axon growth program in the sprouting axons [7]. The increase in GAP-43 expression has been proposed to be stimulated by brain-derived neurotrophin neurotrophic factor (BDNF), which reaches a peak in medial rectus motoneurons after partial deafferentation, several hours before the rise in the density of synaptic terminals [8]. In support of this hypothesis, it is worth noting that both inputs to medial rectus motoneurons express Trk receptors for neurotrophins [17] and, thus, could well be stimulated by BDNF derived from motoneurons. In fact, neurotrophic factors have already been reported to increase not only in response to partial deafferentation [18,19], but also in the formation of new terminal axon branches [20,21].

In the present work, our aim was to test the relevance of BDNF as a regulator of reactive synaptogenesis in the oculomotor system and to probe the retrograde and likely transynaptic communication pathway from the muscle to the motoneuron afferents. For this purpose, partial deafferentation of the medial rectus motoneurons was carried out by sectioning the ATD and BDNF that were injected into the ipsilateral medial rectus muscle to check possible influences of the retrogradely transported molecule. In addition, we were interested in evaluating the ability of BDNF to activate terminal axon sprouting even in the absence of any injury and, for this purpose, the neurotrophin was injected into the medial rectus muscle of naïve animals.

## 2. Results

### 2.1. Increase in Synaptic Terminal Density in the Oculomotor Nucleus after BDNF Was Injected into the Medial Rectus Muscle

**Figure 1:**
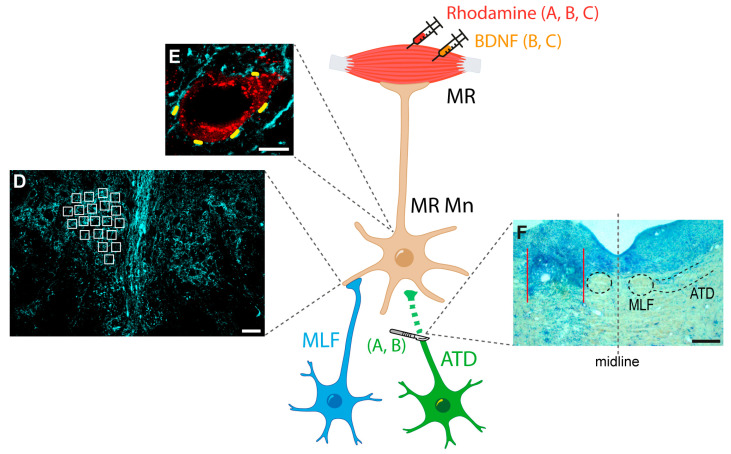
Schematic representation of the experimental approach. Rhodamine was injected bilaterally into the medial rectus (MR) muscles of all animals for the identification of motoneurons. Then, animals were divided into three groups: (**A**) ATD group, with left ATD transection; (**B**) ATD + BDNF group, in which lesioned animals received a dose of BDNF in the left MR muscle; and (**C**) BDNF group, in which naïve animals were injected with the same dose of BDNF in the left MR muscle. In all animals, immunocytochemistry against calretinin was used for terminal identification of medial longitudinal fascicle (MLF), and both low (**D**) and high (**E**) resolution analyses were performed for the synaptic density comparison between groups. The synaptic density at the neuropil level was measured as the optical density inside the regions of interest, spread over the nucleus (white squares in (**D**)). Synaptic coverage around the soma was measured as the percentage of the soma perimeter surrounded by calretinin-positive terminals (short yellow lines around the motoneuron soma in (**E**)). Lesions were evaluated by Nissl staining in sections obtained at the site ((**F**), area between red lines), where intact MLF was observed ((**F**), enclosed by a dashed circle). Right side of the picture (right of the grey stippled line) shows the intact MLF and ATD, encircled and delimited by dashed lines, respectively. Calibration bars: 50 µm in (**D**), 10 µm in (**E**), and 200 µm in (**F**). Abbreviations: ATD, ascending tract of Deiters; BDNF, brain-derived neurotrophic factor; MR Mn: medial rectus motoneurons.

As indicated above, calretinin is specifically expressed by the two pontine populations that innervate medial rectus motoneurons in the oculomotor nucleus: abducens internuclear neurons and lateral vestibular nucleus neurons, whose axons project through the MLF and the ATD, respectively [7,22]. For this reason, immunocytochemistry against calretinin is a good tool not only to visualize afferents to medial rectus motoneurons, but also to quantify and compare the degree of innervation between different experimental treatments and, thus, it also allows us to demonstrate a possible terminal axon sprouting in response to certain perturbations. In particular, following ATD lesion (Figure 1F, area between red arrows in a Nissl-stained section), calretinin-positive synaptic terminals will belong to axons arriving from the MLF.

Under this assumption, we evaluated synaptic density in the neuropil of the rhodamine-identified medial rectus subdivision of the oculomotor nucleus, quantifying the intensity of calretinin immunoreactivity, measured as optical density.

**Figure 2:**
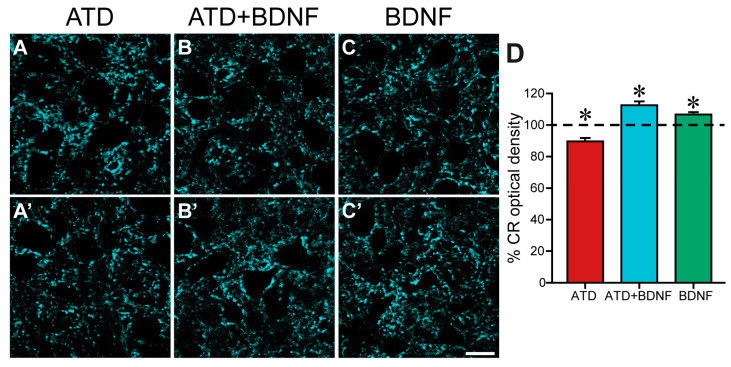
Images showing the immunoreactivity against calretinin, pseudo-coloured in cyan, in the neuropil of the oculomotor nucleus of an animal with unilateral ATD transection (**A**,**A’**), an animal with ATD transection and BDNF injection in the ipsilateral medial rectus muscle (**B**,**B’**), and a non-lesioned animal with BDNF injection in the left medial rectus muscle (**C**,**C’**). In the three cases, the control (**A**–**C**) and the respective treated side (**A’**–**C’**) of the same histological section are shown. Note the lower calretinin immunostaining in the left side (**A’**) of the lesioned animal in comparison with the control side (**A**). In contrast, a higher immunoreactivity against calretinin can be appreciated in the treated side (**B’**,**C’**) as compared with the control side of the ATD + BDNF (**B**) and the BDNF (**C**) animals, respectively. (**D**) Histogram showing mean ± SEM of optical density of the percentage of calretinin (CR) in the neuropil with respect to the control side in the same section. The experimental groups correspond to: ATD (lesion of the ATD), ATD + BDNF (lesion of the ATD + administration of BDNF), and BDNF (non-lesioned animals administered with BDNF). The horizontal dashed line indicating 100% refers to the control side of each section, used for normalised data. Asterisks indicate significant differences with respect to all other groups [*p* < 0.001; *n* (ATD) = 445; *n* (ATD + BDNF) = 340; *n* (BDNF) = 260]. One-way ANOVA followed by post hoc Duncan´s method. Calibration bar for (**A**–**C’**): 20 µm.

Figure 2 shows low magnification images of the neuropil of the oculomotor nucleus immunostained against calretinin, taken from an animal with a lesion in the ATD (Figure 2A,A**’**), an animal with a lesion in the ATD and an injection of BDNF in the medial rectus muscle (Figure 2B,B**’**), and an animal with only a BDNF injection in the medial rectus muscle (Figure 2C,C**’**). A reduction in overall intensity of staining was evident four days after lateral vestibular neuron axotomy in the ATD, when terminal axons, distal to the site of the injury, had already degenerated [8] (Figure 2A, control side, and 2A side of the same histological section). Interestingly, lesioned animals treated with BDNF not only did not show a reduction in calretinin density, but presented a denser calretinin staining in the neuropil of the oculomotor nucleus (Figure 2B’) when compared with the control side (Figure 2B), pointing to an increase in the degree of innervation from the non-lesioned MLF input.

BDNF was also injected into non-lesioned animals to evaluate its capability to stimulate afferent axonal sprouting over motoneurons when the circuit is intact. Under these conditions, motoneurons have not undergone any reaction in response to partial deafferentation, which produces changes in the electrical activity and protein expression of the motoneuron and an altered muscle contraction pattern [7]. A slight increase in calretinin staining could also be detected, as shown in Figure 2C (treated side) as compared to the control side (Figure 2C) of the same section.

Quantitative analysis confirmed the qualitative observations. Since all values were normalized to the mean control value obtained in each section, the mean control optical density in each group was calculated, resulting, respectively, in 100.00 ± 2.19%, *n* = 443 ROIs for the axotomized group; 100.00 ± 1.35%, *n* = 380 for the axotomized group and BDNF group; and 100.00 ± 1.25%, *n* = 360 for the BDNF group. Four days after lesion, calretinin optical density was reduced with respect to its control by 10.4% to 89.6 ± 2.12% in the ATD group (*n* = 445; Figure 2D, red column), which was significantly lower than the mean values obtained in all the other groups either control or treated (one-way ANOVA, Duncan´s method for pairwise multiple comparisons, F_(5,2162)_ = 15.87; *p* < 0.001). Opposing this result, the neuropil of the oculomotor nucleus in lesioned animals with a dose of BDNF (ATD + BDNF group) presented a 12.52% increase in the mean value of optical density (112.52 ± 2.45%; *n* = 340; Figure 2D, blue column) when compared with the control, which was statistically higher than that obtained from all the other groups either control or treated (one-way ANOVA, Duncan´s method for pairwise multiple comparisons, F_(5,2162)_ = 15.87; *p* < 0.001). These results are indicative of an activation of terminal axon growth in abducens internuclear neurons, stimulated by BDNF.

Surprisingly, medial rectus motoneuron afferents also showed a certain degree of sprouting in non-lesioned animals in response to the sole BDNF injection, with a 6.65% increase in calretinin optical density (106.65 ± 1.43%; *n* = 260; Figure 2D, green column) when compared to the control, which, again, was different to all the other groups (one-way ANOVA, Duncan´s method for pairwise multiple comparisons, F_(5,2162)_ = 15.87; *p* < 0.001). This increase in calretinin optical density indicates that MLF and/or ATD axons would be reactive to exogenously administered BDNF from the muscle, even in the absence of ATD motoneuron deafferentation.

### 2.2. Changes in Synaptic Coverage around the Soma of Medial Rectus Motoneurons

**Figure 3:**
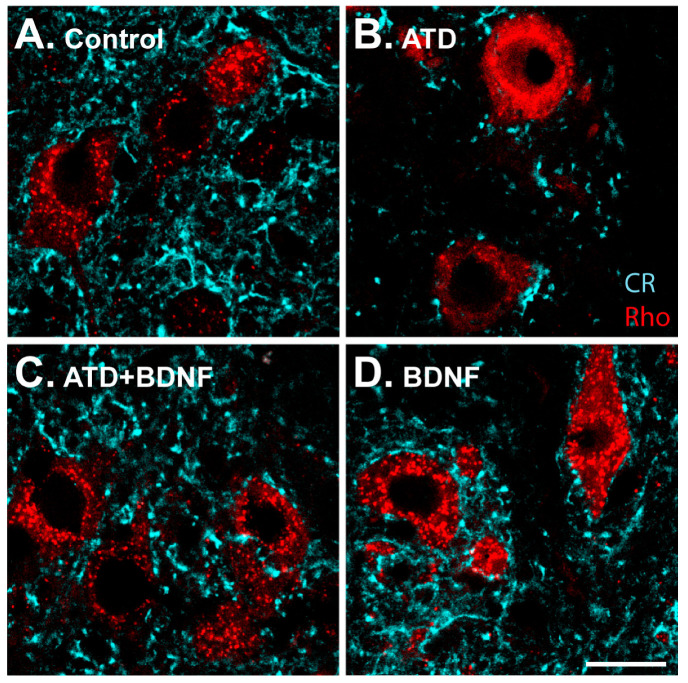
Images showing calretinin-positive terminals (pseudocoloured in cyan) around rhodamine-identified medial rectus motoneurons (red) from control (**A**); from the experimental sides of an animal four days after ATD transection (**B**); an animal four days after ATD transection and injection of BDNF in the ipsilateral medial rectus muscle (**C**); and an uninjured animal with BDNF injection in the left medial rectus muscle (**D**). Calibration bar for (**A**–**D**): 20 µm.

High-magnification images allowed for a detailed analysis of the synaptic coverage around the soma of medial rectus motoneurons (Figure 3). The ATD transection produced, four days later, a drastic decrease in calretinin-positive terminals around the motoneuron somata, in agreement with previous findings [8]. There was a clear reduction in immunopositive puncta (Figure 3B), as compared to the control (Figure 3A), which was not observed in animals injected with BDNF in the medial rectus muscle (Figure 3C,D).

**Figure 4:**
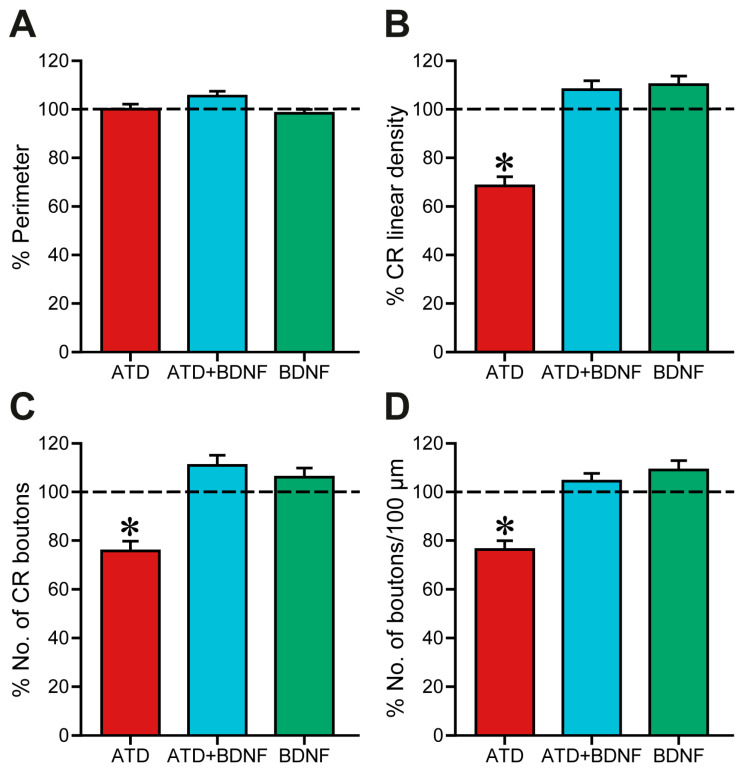
Quantitative analysis of synaptic coverage around medial rectus motoneuron somata. Data are represented as mean ± SEM and expressed as the percentage of the mean obtained with respect to control data of the same experimental group. The horizontal dashed line indicating 100% refers to the control side of each section, used to normalise data. (**A**) No statistical differences in the soma perimeter were found between the groups. (**B**) Synaptic coverage, measured as the percentage of the soma perimeter covered by calretinin-positive terminals. There was a significant reduction after partial deafferentation due to ATD transection (ATD; *p* < 0.001, *n* = 72; asterisk) compared to control, which was not observed after ATD + BDNF treatment (ATD + BDNF; *n* = 86) or BDNF treatment alone (BDNF; *n* = 105). (**C**) Number of calretinin-immunostained terminals around motoneuron soma expressed as percentage of the control side. Partially deafferented motoneurons (ATD) presented a significant reduction in the number of synaptic boutons (*p* < 0.001) when compared with control, ATD + BDNF, and BDNF groups [*n* (ATD) = 118; *n* (ATD + BDNF) = 74; *n* (BDNF) = 36]. (**D**) Number of terminals per 100 µm of somatic perimeter expressed as percentage relative to the control side. Similarly, ATD transection produced a significant decrease in mean values (*p* < 0.001) when compared to the other groups. Neither the deafferented motoneurons with BDNF supply (ATD + BDNF group) nor the intact BDNF-treated motoneurons (BDNF group) showed any difference with respect to control [*n* (ATD) = 72; *n* (ATD + BDNF) = 86; *n* (BDNF) = 105]. For all measurements, one-way ANOVA followed by post hoc Duncan´s method was used. Asterisks: significant differences with respect to the other groups.

Quantitatively, firstly, the soma perimeter was measured to avoid possible biases (Figure 4A). After data normalisation to control mean value (Figure 4A), no changes due to partial deafferentation of medial rectus motoneurons or BDNF injection were detected (one-way ANOVA, control data represented as a horizontal dashed line; red column, ATD-transection group, 100.06 ± 2.03%, *n* = 72 cells; blue column, ATD + BDNF group, 105.39 ± 2.00%, *n* = 86; green column, BDNF group, 98.36 ± 1.58%, *n* = 105). Therefore, changes in the percentage of soma perimeter covered by calretinin-positive boutons could only be attributed to changes in the number, the length, or both, of these boutons.

In fact, four days after partial deafferentation, synaptic coverage (Figure 4B, red column), measured as the percentage of soma perimeter covered by calretinin-immunoreactive puncta, was decreased to 68.42 ± 3.73%, which was statistically different when compared with the control, ATD + BDNF, and BDNF groups (one-way ANOVA, Duncan´s method for pairwise multiple comparisons, F_(5,487)_ = 16.33; *p* < 0.001). Normalised synaptic coverage of both partially deafferented motoneurons supplied with BDNF (108.06 ± 3.69%, Figure 4B, blue column) and intact motoneurons after injection of BDNF (110.14 ± 3.5%, Figure 4B, green column) was not changed compared with control data (100.00 ± 2.93%, 100.00 ± 3.09%, 100.00 ± 5.82%, for axotomized animals, axotomized + BDNF, and BDNF animals, respectively; Figure 4B).

Analysis of the total number of calretinin-positive boutons around each motoneuron soma offered similar results (Figure 4C). Again, ATD transection produced a decrease of 75.72 ± 4.03% in the number of ATD terminals (one-way ANOVA, Duncan´s method for pairwise multiple comparisons, F_(5,487)_ = 8.59; *p* < 0.001), a reduction in terminals that was prevented when BDNF was injected into the muscle (110.89 ± 4.16%), likely originating from the MLF. Intact motoneurons provided with an additional supply of BDNF exhibited a total number of terminal boutons that was similar to the control (106.01 ± 3.81%).

Furthermore, the number of boutons per 100 μm of soma perimeter was calculated and normalised with respect to the mean control value obtained in the animals of the same experimental group (Figure 4D). Consistent with previous analyses, four days after ATD transection a reduction of 76.3 ± 3.63% was observed, which was statistically lower than in the other groups (one-way ANOVA, Duncan´s method for pairwise multiple comparisons, F_(5,485)_ = 9.36; *p* < 0.001). No changes were observed with respect to control after either ATD + BDNF treatment or with BDNF alone. Mean bouton length was also calculated but no changes were detected between groups.

Altogether, these results in somatic coverage indicate that, first, four days after ATD transection there was an overall 75% decrease in the degree of innervation around medial rectus motoneuron somata. Second, ATD transection plus injection of BDNF into the medial rectus muscle, the target of partially deafferented motoneurons, prevented this decrease in innervation. Given the ATD deafferentation, this could imply that the remaining calretinin-positive afferents would have sprouted new branches and terminal boutons, possibly stimulated by the activity of the retrogradely and transynaptically transported BDNF. Third, the injection of BDNF into naïve animals did not activate any sprouting signal in either MLF or ATD terminals around the soma.

## 3. Discussion

**Figure 5:**
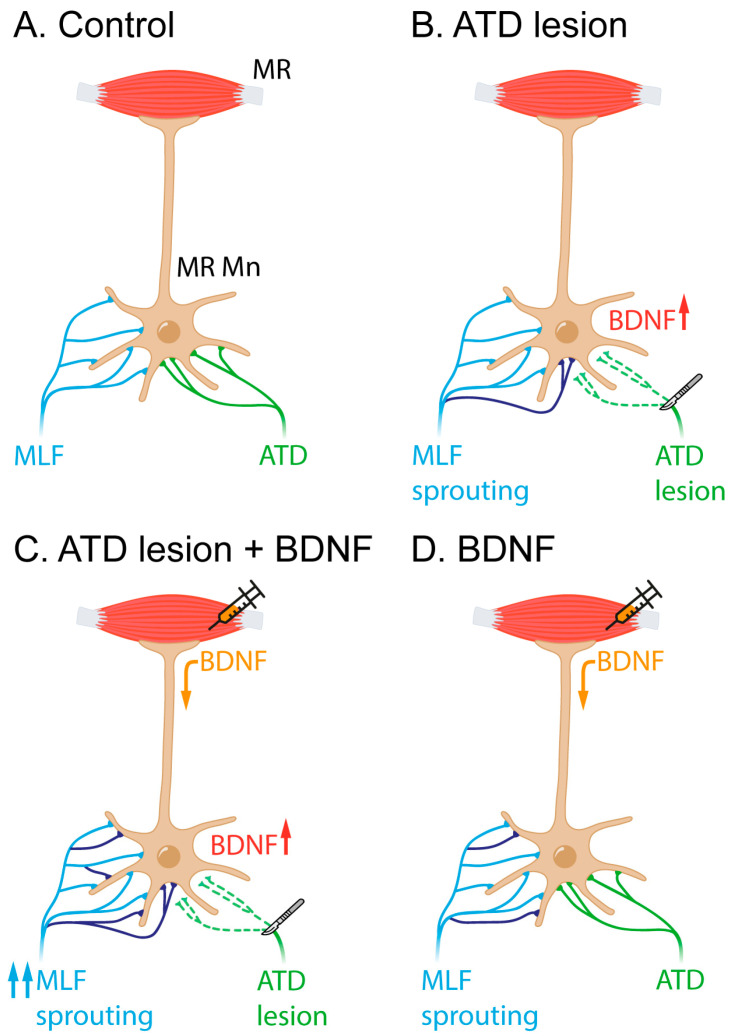
Schematic representation of the interpretation of the present results. (**A**) In the control situation, medial rectus motoneurons (MR Mn) receive input from the MLF (in blue) and the ATD (green). The ATD projection is less exuberant than the MLF projection and terminates preferentially on the soma of the motoneurons in contrast to the MLF input, which ends mostly in the dendritic compartment. (**B**) Four days post-ATD section, an increase in BDNF, among other molecular signals, might induce MLF sprouting (newly originated branches and terminals shown in purple). (**C**) When BDNF is exogenously provided by the muscle, the ATD lesion produces a more intense sprouting from the MLF (purple). (**D**) BDNF injection in naïve animals also produces sprouting to some extent, likely originating from the MLF (purple).

The main objective of this work was to elucidate whether the neurotrophin BDNF plays any role in the activation of the sprouting, during the reactive synaptogenesis, that occurs after partial deafferentation of motoneurons. For this purpose, two experiments were carried out. First, we unilaterally lesioned the ATD of adult rats, to partially remove the afferents to the medial rectus motoneurons. In this respect, our previous study [8] has shown that during the first four days after lesion, the synaptic coverage of partially deafferented motoneurons diminishes, reaching a minimum 48 h after lesion. The number of terminals then increases, with significant differences 96 h after lesion. This finding was explained as the result of the sprouting of the remaining afferents, mainly the terminal axons from the abducens internuclear neurons that course through the MLF. However, synaptic coverage did not recover control levels [8]. In this work, we aimed to check whether an injection of BDNF into the medial rectus muscle, the target muscle of the partially deafferented motoneurons, performed immediately after lesion, could boost the reactive synaptogenesis (Figure 5). In fact, this was the case, as we demonstrated that synaptic coverage in the neuropil, i.e., on the dendrites of medial rectus motoneurons, not only resumed to control values but surpassed them. In the case of the synaptic coverage around motoneuron soma, all parameters returned to control values. This could be interpreted as meaning that the intact MLF afferent system, composed by the fibres originating in the abducens nucleus, had undergone terminal sprouting to occupy the vacant synaptic space left after ATD axon removal. Most significantly, we showed that BDNF addition increases this sprouting.

The second experiment consisted of the injection of BDNF into the medial rectus muscle of naïve animals, to check whether BDNF could, by itself, activate terminal sprouting even in the absence of lesion. Results show that this was the case in the neuropil, but not around the soma. As argued below, this probably means a differential effect of BDNF on abducens internuclear neurons vs. lateral vestibular nucleus neurons.

### 3.1. Effect of Exogenous BDNF Supply on Reactive Synaptogenesis after Partial Deafferentation

Reactive synaptogenesis has been described to occur when a given neuronal population partially losses its afferents. As a result, remaining inputs sprout and occupy the vacant synaptic places left [1,2,3,4,5,6]. This is also the case for medial rectus motoneurons that receive two major inputs, which is an amenable system for producing a partial deafferentation. In fact, when the ATD input is eliminated, a transient loss of the characteristic firing pattern has been described in medial rectus motoneurons during the first days after lesion [7], although these changes eventually return to normality. Accordingly, a reduction in the synaptic coverage around the medial rectus motoneuron soma and dendrites was described 24 h after partial deafferentation, reaching a minimum 24 h later, and then recovering to an intermediate level between the minimum and the control situation 96 h after lesion [8]. This timing defines a wave of synaptic degeneration followed by terminal sprouting of a different system of axons that contributes to maintaining the network properties of deafferented neurons. A similar cycle of degeneration and regeneration has been found in other neuronal systems [23]. The consequences of terminal sprouting are generally thought of as a form of homeostatic plasticity that tends to maintain the firing properties of neurons [7], although there is not always a complete correlation between the extent of terminal sprouting and the behavioural recovery from the lesion [24]. In the present work, an injection of BDNF was performed into the ipsilateral medial rectus muscle, during the same surgical session as the ATD lesion. This additional trophic supply boosted the recovery of synaptic coverage by a different degree depending on the location under study: recovery at the neuropil exceeded control values, while synaptic coverage around the motoneuron soma matched control data. This is interesting, because the two inputs to medial rectus motoneurons, abducens internuclear and vestibular neuron axons, do not distribute over the same territories on the somato-dendritic membrane of the motoneurons, but rather innervate specifically different neuronal compartments. In particular, abducens internuclear neuron axons end preferentially over motoneuronal dendrites, and vestibular axons terminate primarily over the somatic membrane [16] (Figure 5A). This implies that ATD transection eliminates synaptic input mainly in the soma, but not on the dendrites. This result is evidenced in the present findings, because at four days after lesion, when reactive synaptogenesis has already occurred, the decrease in optical density due to calretinin staining in the neuropil represented 10% of the control, while there was a reduction of approximately 30% around the soma (Figure 5B). It is important to note that the new terminals are immunoreactive to calretinin, which implies that they originate in the abducens nucleus. When BDNF was retrogradely supplied, a total recovery of all synaptic coverage was observed around the soma, indicating, first, the capability of the MLF axons to spread from the dendritic to the somatic compartment to fill the vacant synaptic spaces left after removal of ATD and, second, the fact that BDNF improves this ability (Figure 5C). These findings are consistent with previously reported results: (i) BDNF protein increases in the medial rectus motoneuron soma 48 h after partial deafferentation [8], as described for other motoneurons [18] and cortical neurons [19]; (ii) adult abducens internuclear neurons express TrkB, the specific receptor for BDNF [17]; and (iii) BDNF has been shown to increase axonal GAP-43 expression [25], thus potentiating axonal sprouting. Many works show the direct role of BDNF on terminal sprouting and general restoration (see for review [26]). For instance, targeting BDNF directly at phrenic motoneurons by means of adeno-associated virus has been shown to restore the diaphragm activity by promoting the growth of ventral respiratory group afferent axons [27]. On occasion, the terminal sprouting caused by BDNF overexpression might result in maladaptive exacerbation of undesired network responses [28]. Altogether, it could be suggested that partial deafferentation of medial rectus motoneurons would produce an increase in BDNF, which, eventually, would be released through the somatodendritic membrane of the motoneurons and then bind to the TrkB receptors present in the presynaptic membrane of abducens internuclear neurons. Therein, BDNF-TrkB signalling might activate the sprouting process of reactive synaptogenesis, probably through the activation of the synthesis of GAP-43. Axons would then grow and form new branches, ending as terminal boutons over medial rectus motoneuron membrane. The BDNF in the motoneurons could have originated from the motoneuron itself, but the present results point to the muscle as the origin of the trophic molecule, as we tested the retrograde pathway of BDNF in our experiments. In line with our findings, it has been shown that fatigue resistance, as a specific feature of some muscles, including the extraocular muscles, depends on the activity-dependent secretion of some neurotrophic factors such as BDNF and NT-4 [29].

The results in the dendritic compartment are noteworthy. After partial deafferentation, BDNF not only prevented the fall in calretinin-positive puncta, but the data exceeded those obtained in the control situation. This result implies that abducens internuclear neurons, once stimulated by this trophic factor, may even form new synapses in new places, and not only in those left vacant after partial deafferentation.

These results are interesting from a clinical point of view: amyotrophic lateral sclerosis is a neurodegenerative disease in which cortical and spinal motoneurons degenerate, a process that in a period of less than five years leads to respiratory failure and death. Degeneration onset varies among patients, some of them starting with lower motoneuron death, while some others start with cortical degeneration (for review, see [30]). Motor impairment would then start when this upper death results in lower motoneuron death, probably due to changes derived from the reduction in afferent activity. The discovery of muscle-derived BDNF as an inductor of sprouting after partial deafferentation could, then, be thought of as a potential therapy to stop, or at least delay, spinal motoneuron death, as has already been shown in murine models of amyotrophic lateral sclerosis [31]. In line with this, BDNF-dependent axonal transport has been found to be defective in the mouse model of amyotrophic lateral sclerosis SOD1 [32].

### 3.2. Effect of Exogenous BDNF Supply on Synaptic Coverage in Intact Motoneurons

The fact that the injection of BDNF into the medial rectus muscles of naïve animals had an impact on the synaptic coverage over motoneuron dendrites, but not in the soma compartment, is intriguing (Figure 5D). The two afferent systems, abducens internuclear neurons and vestibular neurons, have a distinct distribution over their target population: while the former mainly end in the dendritic compartment, the latter are distributed preferentially over the soma [16]. Nevertheless, BDNF might have influenced the two afferent systems to form new axonal branches in the motoneuron dendrites. In fact, both populations express TrkB, the specific receptor for BDNF [17] and, thus, both could react in a similar way to the retrograde supply of BDNF. However, the vestibular projection is much weaker (in terms of axon terminal density) than that arising from the abducens nucleus. Thus, a much larger projection of internuclear neurons would mean a much larger availability of TrkB receptors, and a higher responsiveness to BDNF supply. Furthermore, the ATD projection terminates preferentially in motoneuron somata, and our findings did not show any increase in the somatic terminals. In fact, Hernández et al. [7] showed a lower functional recovery of medial rectus motoneurons after partial deafferentation when the ATD was transected, in comparison with the effects after MLF transection. If, indeed, recovery was due to axon sprouting from the remaining afferents, then it could be possible that a less BDNF-responsive afferent system could restore function to a lesser degree.

The effect of BDNF injection was higher in partially deafferented motoneurons when compared with intact ones. This could indicate that, although BDNF seemed to be sufficient to trigger the activation of the sprouting machinery, there must be other factors involved that boost the process. These other factors may range from other target-derived neurotrophins [33,34], to immune-related molecules such as Il-6 [35], and complement proteins [36] that could act through the protein kinase C route [37]. The precise nature of these factors should be analysed.

## 4. Materials and Methods

A total of 10 male Wistar rats, weighing 250–300 g, were divided into three different groups: (i) animals used to perform the lesion of the ATD (*n* = 4); (ii) animals lesioned in the ATD and injected with BDNF in the ipsilateral medial rectus muscle (*n* = 3); and (iii) non-lesioned animals receiving the BDNF injection (*n* = 3) (Figure 1A–C). All experimental procedures were revised by the local ethics committee and followed the European Union Directive on the protection of animals used for scientific purposes (2010/63/EU), and the Spanish legislation (R.D. 55/2013).

### 4.1. ATD Transection

ATD was sectioned to produce a partial deafferentation of medial rectus motoneurons. Deeply anesthetized animals (ketamine + xylacine 80 + 10 mg/kg, i.p., after a protective atropine injection of 0.05 mg/kg, i.m.) were placed in a stereotaxic frame. The skull was exposed along the parietal bone suture and a hole was drilled in the occipital bone. After bregma identification, a custom-made 0.5 mm-wide blade was situated with the right corner in the midline and guided using a micromanipulator towards the appropriate coordinates [38]: 13 mm posterior to bregma, 11 mm deep, 0.15 mm to the left from the midline and using an anterior angle of 30 degrees. Therefore, ATD transection was carried out unilaterally on the left side. Skin was sutured and animals were monitored until complete recovery.

### 4.2. BDNF Injection

We were interested in the following aims regarding BDNF: (i) to evaluate the capability of BDNF to stimulate terminal axon sprouting; (ii) to elucidate whether BDNF is a sufficient factor to produce this activation; and (iii) to determine the possible origin of the increment in BDNF detected in partially deafferented medial rectus motoneurons [8]. For these purposes, 1 µL of BDNF (Merck, Darmstadt, Germany) at 1 µg/µL in phosphate buffer saline (PBS) was injected under deep anaesthesia with a 34-gauge Hamilton syringe (Reno, NY, USA) in the left medial rectus muscle of either intact or ATD-transected animals (Figure 1B,C; two experimental groups).

### 4.3. Retrograde Labelling of Motoneurons

All animals were injected with rhodamine (rhodamine B isothiocyanate; Sigma, St. Louis, MO, USA) in the medial rectus muscle for retrograde identification of motoneurons. Medial rectus muscles were bilaterally injected with 1 µL of 20% rhodamine prepared in a solution of 2% dimethylsulfoxide in PBS, using a Hamilton syringe (Figure 1A–C; the three experimental conditions).

All surgical procedures and injections were carried out sequentially for each animal during the same session.

### 4.4. Immunohistochemistry

Four days (96 h) after rhodamine injection, deeply anesthetized animals (sodium pentobarbital, 100 mg/kg, i.p.) were perfused transcardially with 0.9% saline, followed by 4% paraformaldehyde in phosphate buffer 0.1 M, pH 7.4. Their brains were then dissected and cryoprotected in 30% sucrose prepared in phosphate buffered saline (PBS). Then, 40-µm-thick brainstem coronal sections were cut using a cryostat and collected in glycerol-PBS (1:1) for storage at −20 °C.

For the study of the effect of partial deafferentation and BDNF administration on axon terminal sprouting from abducens internuclear neurons, we used immunofluorescence against calretinin. Briefly, sections were rinsed, blocked with 7% normal goat serum (NGS) in PBS with 0.1% Triton X-100 (PBS-T), and incubated in the primary antibody (rabbit anti-calretinin, 1:5000; Swant, Burgdorf, Switzerland) at room temperature for 12 h. Sections were then washed and incubated for 2 h in the secondary antibody solution (goat anti-rabbit IgG coupled to Cy5, 1:200 in PBS-T; Jackson Immunoresearch, West Grove, PA, USA). After several washes in PBS, sections were mounted on glass slides and coverslipped with DakoCytomation (Dako, Glostrup, Denmark).

### 4.5. Nissl Staining

Brainstem sections from the ponto-mesencephalic region were selected from lesioned tissue for Nissl staining to visualize the lesion and assess its adequate localization. Briefly, sections were mounted on gelatinized slides and left for 24 h to dry. Slides were then immersed in a solution containing Toluidin blue 0.5% (Sigma-Aldrich) diluted in acetic-acetate buffer (pH 4.2), washed in distilled water, and dehydrated through successive immersions in alcohol 70%, 80%, 90%, and 100%. Tissue was then immersed in xylene and coverslipped with DPX.

### 4.6. Image Analysis

The oculomotor nucleus was visualized using a Zeiss LSM 7 Duo confocal microscope (Zeiss, Oberkochen, Germany). The lasers DPSS 561 nm and HeNe 633 nm were used to excite rhodamine and Cy5, respectively.

To evaluate the effect of each treatment on axon terminal sprouting in the neuropil of the medial rectus subdivision of the oculomotor nucleus, 6 × 3 tile images at ×40 magnification were obtained from each section, captures were stitched, and both sides of the nucleus were made visible in the same image. Medial rectus motoneurons were located by their retrograde staining with rhodamine. The intensity of calretinin immunostaining in the neuropil, measured as optical density, was analysed using ImageJ software (NIH, Bethesda, MD, USA). A total of 20 randomly selected square regions of interest (ROIs) were selected on each side of the same histological section, control, and treated (Figure 1D). The background level of staining was determined as the mean of the optical density in three different ROIs and selected inside the soma of three different motoneurons, as they were completely negative for calretinin. The signal obtained within each ROI was divided by the mean background signal. The neuropil optical density was normalised to the control side and expressed as a percentage. Thus, a mean signal value was calculated for each section on the control side, and all optical density values in that section (control and treated) were recalculated as a percentage of the mean control value. The average of these percentages was calculated for each experimental group. For neuropil measurements, six groups (three experimental groups + their respective control sides) were compared using one-way analysis of variance (ANOVA) followed by Duncan’s method for multiple pairwise comparisons, at a level of significance of *p* < 0.05, performed with SigmaPlot software, version 11.0 (Systat Software, Inc., San Jose, CA, USA).

The analysis of synaptic coverage around medial rectus motoneuron somata was also performed (Figure 1E). For this purpose, each section was visualized under a ×63 oil-immersion objective, and z-stack images (2 µm virtual thickness) were obtained. ImageJ software was then used to measure soma perimeter and the length of each individual terminal, and to count the number of terminals around the perimeter of each soma, the relative length of the perimeter occupied by axon terminals (expressed as the percentage of the total perimeter covered by calretinin-positive terminals), and the number of boutons per 100 µm of perimeter. For each experimental condition, data were collected from both sides of each section, i.e., control and treated, and normalised with respect to the mean control value obtained from the same experimental group of animals (i.e., a total of 6 groups, 3 control and 3 experimental, obtained from both sides of the same histological sections). Therefore, control data were obtained from the control side of each experimental group. These data were expressed as mean ± SEM and compared with a one-way ANOVA test (SigmaPlot 11, San Jose, CA, USA), with an overall significance level of 0.05. Post hoc tests used Duncan´s method for multiple comparisons. For the quantitative graphs of measurements performed both in the neuropil and in the cell bodies, control data (from the three experimental groups) were illustrated as a dashed line at 100% value for clarity.

## Data Availability

Data are available upon kind request.
